# Daylight affects human thermal perception

**DOI:** 10.1038/s41598-019-48963-y

**Published:** 2019-09-23

**Authors:** Giorgia Chinazzo, Jan Wienold, Marilyne Andersen

**Affiliations:** Laboratory of Integrated Performance in Design (LIPID), School of Architecture, Civil and Environmental Engineering (ENAC), Ecole polytechnique fédérale de Lausanne (EPFL), 1015 Lausanne, Switzerland

**Keywords:** Sensory processing, Human behaviour, Civil engineering

## Abstract

Understanding the factors that affect human thermal responses is necessary to properly design and operate low-energy buildings. It has been suggested that factors not related to the thermal environment can affect thermal responses of occupants, but these factors have not been integrated in thermal comfort models due to a lack of knowledge of indoor factor interactions. While some studies have investigated the effect of electric light on thermal responses, no study exists on the effect of daylight. This study presents the first controlled experimental investigation on the effect of daylight quantity on thermal responses, combining three levels of daylight illuminance (low ~130 lx, medium ~600 lx, and high ~1400 lx) with three temperature levels (19, 23, 27 °C). Subjective and objective thermal responses of 84 participants were collected through subjective ratings on thermal perception and physiological measurements, respectively. Results indicate that the quantity of daylight influences the thermal perception of people specifically resulting in a cross-modal effect, with a low daylight illuminance leading to a less comfortable and less acceptable thermal environment in cold conditions and to a more comfortable one in warm conditions. No effect on their physiological responses was observed. Moreover, it is hypothesised that a warm thermal environment could be tolerated more whenever daylight is present in the room, as compared to the same thermal condition in a room lit with electric lights. Findings further the understanding of factors affecting human thermal responses and thermal adaptation processes in indoor environments and are relevant for both research and practice. The findings suggest that daylight should be considered as a factor in thermal comfort models and in all thermal comfort investigations, as well as that thermal and daylight illuminance conditions should be tuned and changed through the operation and design strategy of the building to guarantee its occupants’ thermal comfort in existing and future structures.

## Introduction

An accurate control and design strategy for the indoor thermal environment is necessary to improve the comfort, well-being and general health of building occupants, as well as to boost their work performance^1,2^, and limit building energy consumption^3,4^. Conventionally, the operation of air-conditioning systems has been based on the *heat balance model* for thermal comfort developed in the 1970s for air-conditioned buildings^5^ and extended later for non-air-conditioned buildings in warm climates^6^. The model predicts the human thermal sensation by generating a numeric value called the “predicted mean vote” (PMV) that ranges from cold to hot as a function of physical parameters (i.e., air temperature, mean radiant temperature, air velocity and humidity) and of personal factors (i.e., metabolic rate and clothing insulation). Another well-known model suggested for the design and control of the thermal environment in non-air-conditioned buildings (i.e., naturally ventilated), is based on adaptive theory. Adaptive theory considers occupants as active users who enact changes to their thermal environment via behavioural adjustments (e.g., opening windows and use of blinds and fans), physiological adaptation (i.e., acclimatization) and psychological expectation and habituation in relation to the indoor and outdoor environment^7–9^. Based on the adaptive theory, the standardised *adaptive model*^10–12^ relates the acceptable ranges of indoor temperature to outdoor temperatures, supposing an expectation and habituation process, and is derived from data mined in naturally ventilated buildings from field studies (i.e., an *in situ* polls on comfort evaluations together with measurements of the actual indoor conditions)^13^.

The application of the adaptive model instead of the heat balance model allows for an extended acceptable range of temperatures in buildings, resulting in substantial reduction of energy consumption^14^. In addition, the adaptive model could help to explain the gap observed in field studies between the thermal sensation vote reported by people (i.e., “actual sensation vote”, asv) and the calculated PMV^7,15^. To further expand the temperature range to be used in buildings and reduce the asv-PMV gap (i.e., “actual sensation vote” - “predicted mean vote” gap), other types of adaptation processes have been proposed as an extension of the current adaptive model or as integration to the heat balance model^16–18^. To date, despite having been identified as likely influencing parameters of occupants’ thermal sensation^7^, the indoor environmental parameters not related to thermal conditions (i.e., light, acoustic ambiance and air quality) have never been included in thermal comfort models.

The thermal sensation predicted by thermal comfort models is just one of the possible “thermal responses” that could result from an environmental stimulus. In the context of this research, we consider thermal sensation as part of the “subjective thermal perception”, together with thermal comfort, preference and acceptability. Researchers have suggested that thermal sensation is the first subjective response to the physical environment^19^, and that it is strongly correlated with physiological responses^20^. On the other hand, thermal preference, comfort and acceptability have been considered to be the results of the reflection upon the sensation, i.e., they are thermal evaluations^19^. The relationship between thermal sensation and thermal evaluations has been explained physiologically with the concept of *alliesthesia*^21,22^ and psychologically^19^ relying on expectations, adaptations and other factors^7,19,20,23^. In this study, together with the aforementioned subjective thermal perception ratings, physiological responses will be evaluated as additional thermal responses.

The simultaneous presence of thermal and non-thermal factors in indoor spaces can result in two different effects: *combined effects* when the overall perception of the indoor environment is affected by the combined presence of multiple indoor factors^16,24–26^, and *cross-modal effects* when thermal responses (intended as both subjective thermal perception and physiological responses) are influenced by factors not related to the thermal environment (i.e., non-thermal factors). Despite the potential application of results on cross-modal effects in all indoor spaces due to the simultaneous presence of multiple environmental stimuli, there is a lack of integration of non-thermal factors into thermal comfort models. This is the consequence of a limited knowledge on the interactions of indoor factors^27^.

Among the possible interactions of indoor factors, the study of the effect of light conditions on thermal responses has been the most investigated^27^, considering the large implications that results could have on energy consumption of buildings^28^ due to the possibility of fine-tuning the thermal environment according to the lighting conditions. In addition, many studies on the cross-modal effect of light on thermal responses have been conducted in relation to non-image forming effects of light^29^, following the discovery of a new photoreceptor in the human eye^30^. In this context, the effect of *electric light quantity* on physiological responses (e.g., core body temperature and skin temperature) has been largely investigated in controlled laboratory experiments^29^. Findings from these studies show that exposure to bright light in the evening affects the human thermoregulation rhythm by delaying the natural decline of the core body temperature while simultaneously slowing down the increase of distal skin temperature^29^. On the other hand, it has been observed that an exposure to bright light in the morning results in a faster increase of core body temperature^29^.

Only a handful of studies have investigated the effect of light on physiological responses together with subjective thermal perceptions^31–36^ or focused on subjective thermal perceptions alone^28,37,38^. As for investigations on physiological responses only, all of these studies investigated the effects of variations of electric light quantity. It has been hypothesised that the light could have an indirect effect on subjective thermal perceptions through variations of human thermoregulation rhythm (observed with changes in physiological responses)^29^. A correlation between time of exposure, light intensity, core body temperature, and subjective thermal sensation was found in some studies that investigated both physiological responses and subjective thermal perception^31–33^. In particular, it has been reported that after a bright light exposure during the daytime, the core body temperature was lower and the thermal sensation was warmer compared to an exposure to dim light. On the other hand, after a bright light exposure at night, the core body temperature was higher and the thermal sensation was colder compared to dim light. A cooler thermal sensation after exposure to dim light compared to bright light was also confirmed in experiments reporting only subjective thermal sensation, with exposure during the day^37^ or at an unknown time of the day^38^. However, contradictory^35^ or inconclusive^28,34,36^ results have been reported in other studies investigating subjective thermal perceptions only. These incongruities can be explained in terms of different experimental design features such as the timing and duration of the light exposure or the light intensity^29^. For example, the use of a too low light intensity for the bright light exposure can lead to inconclusive results^36^. We also suggest that differences between the time of the light exposure and that of the measurement should be considered. In other words, do they correspond? If not, what is the thermal environment in both of them? What is the visual environment? Furthermore, the type of thermal environment should be taken into account (e.g., static vs. dynamic or comfortable vs. not comfortable) and the type of thermal perception investigated (i.e., thermal acceptability was never investigated). Based on these considerations, it is clear that further investigations are necessary for the understanding of the relationship between light, subjective thermal perceptions and physiological responses according to different timing and duration of the light exposure and variations in the thermal environment.

Considering that all previous investigations relied on electric lighting^29,34^, there is a lack of knowledge on how *daylight* affects thermal responses, intended as both subjective thermal perception and physiological responses. Only one study by the authors examined the effect of daylight on subjective thermal perceptions (i.e., thermal sensation and thermal evaluation), but in field studies^39^ where many parameters could not be controlled but only measured, and where physiological measurements were not collected. The use of natural light instead of electric light, besides causing variations in the thermal environment due to physical changes (i.e., heat gains and losses through the glazing), might result in different and additional psychological effects, as it has been reported in previous visual comfort studies^40,41^, especially considering that the concept of light-warmth and darkness-cold is intrinsic to the human psyche^38^. As an example of this psychological effect of daylight on subjective thermal perception, it has been hypothesised that the thermal environment in a naturally-lit space could be more tolerated than what is predicted by the PMV of the heat balance model^42^. Given the large presence of daylight in buildings and the tendency to design highly glazed façades, studying the implications of daylight on thermal responses is becoming even more of an urgent problem to tackle. However, considering the number of confounding factors combined with the inability to control daylight conditions in field studies^39^ (e.g., illuminance levels can only be measured and not controlled or changed, or blinds position are defined by the users), it is necessary to conduct such investigations in a controlled environment if one aims to identify specific effects or interactions with the thermal environment. It must be remarked, however, that in experiments with daylight (even when performed in a controlled environment), not all possible visual parameters can be controlled due to the contact with the outside and the changes of daylight positions over the day and the year. Acknowledging this limitation, in the context of this research, a daylight experiment is defined “controlled” whenever some conditions of the visual environment (e.g., illuminance levels, blinds position, type of view to the outside, colour of the glazing) can be decided, changed and kept (semi-)constant, contrary to field studies.

Addressing the challenges highlighted in the analysed literature, this study reports the results of a controlled experimental study investigating the influence of daylight on thermal responses, intended as both subjective thermal perceptions (i.e., thermal sensation and thermal evaluations) and physiological responses (i.e., skin temperature), by studying different combinations of daylight and temperature levels. As examined for electric light in past studies, different daylight levels (i.e., low, medium and high illuminance levels) were studied to explore their cross-modal effect on thermal responses. In addition, to account for interactions (i.e., to consider under which thermal environment cross-modal effects occur), the same investigation was performed at three temperature levels. This also allowed the study of the combined effects of daylight and temperature levels on overall comfort. It was hypothesised that: (i) thermal responses are affected by daylight, and that they would be lower (i.e., colder and less acceptable for thermal perception) under low illuminance versus high illuminance daylight levels (*cross-modal main effect*); (ii) the cross-modal effect depends on the thermal environment people are exposed to (*cross-modal interaction effect*); (iii) daylight and temperature contribute equally to the evaluation of the indoor environment as a whole (*combined effect*); (iv) the thermal environment in a warm condition is more tolerated when daylight is present compared to the evaluation of the same thermal environment in an electrically-lit space. For the last point, analysis of the consideration of the visual environment into thermal comfort models will be presented. This analysis will focus on the comparisons between the calculated PMV according to measured indoor environment parameters and the metabolic rate and clothing insulation of participants and the thermal sensation rating (asv) reported by participants.

## Methods

### Experimental design

The experiment consisted of a 3 × 3 mixed factorial design with the factor “temperature” as the between-subjects factor and the factor “daylight” as the within-subjects factor. The three temperature levels (19 °C, 23 °C and 27 °C) were chosen to have a comfortable thermal environment (23 °C) and two slightly uncomfortable ones (slightly cold and warm), according to the clothing insulation and the activity level of participants^43^. The three levels of daylight were low, medium and high, and depended on the average maximum daylight level achievable in the experimental test room on a completely sunny day. The daylight levels were presented in a counterbalanced order across participants and temperature levels. Temperature levels were randomised across experimental days.

### Ethics

Written informed consent was obtained previous to the experiment. The study was approved by the Cantonal Ethics Committee of the Canton Vaud, Switzerland (CER-VD, Ref. No. 2016-01115), and adhered to the principles of the Declaration of Helsinki.

### Participants

Volunteers were sought through the subject pool of the Universities in Lausanne (Switzerland) with an email invitation and by advertisements on local billboards. Only people satisfying the inclusion criteria (age 18–30 years old, no abuse of alcohol and use of drugs, full colour vision, generally healthy, French speaking –mother tongue or C2 level, BMI between 18 and 25 kg/m^2^, no visual or motor abnormalities) were invited to take part in the study. People aware of the research aim were excluded from the experiment to avoid response bias. A total of 122 participants took part in the experiment. Due to unpredictable variations in daylight conditions (i.e., clouds in front of the sun and moving in the sky), 19 experimental sessions with two participants each had to be excluded from the analysis, resulting in a final sample size of 84 participants (42 women and 42 men). Each temperature level was experienced by the same number of people, and sex balance was respected. All participants had normal or correct-to-normal vision and none of them was classified as extreme chronotype according to the Morningness-Eveningness Questionnaire (MEQ between 30 and 70)^44^. Table 1 summarises participants’ characteristics for the total sample size and for each temperature level. Participants were paid for taking part in the study. They received a total of 50 CHF at the end of the experiment.

**Table 1:** Participants’ characteristics for the total sample and at each temperature level (mean ± SD, where applicable).

	Total sample	19 °C	23 °C	27 °C
N [−]	84	28	28	28
Sex [−]	50% W, 50% M	50% W, 50% M	50% W, 50% M	50% W, 50% M
Age [years]	19.2 ± 1.3	19.4 ± 1.4	19.3 ± 1.1	19.0 ± 1.3
Weight [kg]	63.0 ± 9.2	64.3 ± 9.2	62.3 ± 8.2	62.6 ± 10.1
Height [m]	1.7 ± 0.1	1.7 ± 0.1	1.7 ± 0.1	1.7 ± 0.1
BMI [kg/m^2^]	21.2 ± 2.3	21.5 ± 2.3	21.0 ± 2.1	21.2 ± 2.4
MEQ [−]	50.0 ± 9.1	51.4 ± 8.5	50.0 ± 9.6	48.5 ± 9.3

### Apparatus and protocol

**Figure 1:**
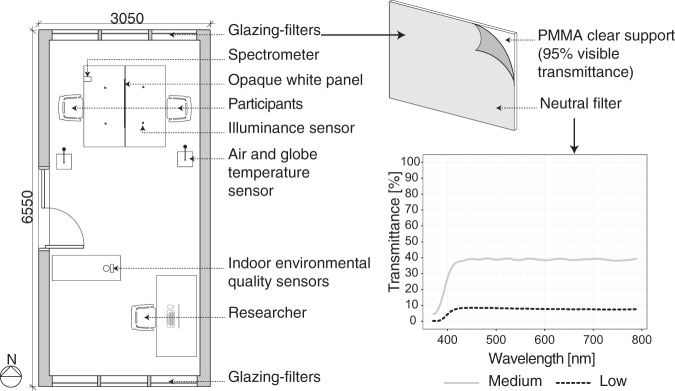
Experimental room floor plan and dimensions, colour neutral filter construction and spectral transmittance (for the low and the medium illuminance conditions).

The study was conducted in an office-like test room (3.05 m × 6.55 m) located on the University campus in direct contact with the external environment (Fig. 1). The room was previously used for other user studies on visual and thermal interactions^45–47^. The test room allowed the researchers to set and control the thermal stimuli through radiant surfaces (capillary tube system placed in high density fibreboard sandwich panels, connected to an external air-to-water heat pump) and a heat-recovery mechanical ventilation system, while the visual factors (linked to daylight) were varied by applying colour neutral filters on the south and the north openings. This method allowed for a constant view to the outside (quantity and quality). Colour neutral filters with two transmittance levels (37% and 9%, see Fig. 1 for spectral transmittance values) were placed on the glazing to reach the medium and the low illuminance daylight levels, respectively. The filters were glued on a transparent PMMA support (95% transmittance) that was then inserted within the windows’ frames and blocked with black wooden bars with magnets. The high illuminance level corresponded to the clear glazing (coated double glazing Pilkington optiwhite^TM^, low iron extra clear float glass) without any additional filter applied on it, with a visual transmittance of the glazing of 75%. The three glazing-filter combinations resulted therefore in a final visual transmittance of 75%, 30% and 7%. A blackout curtain, installed on both openings, blocked daylight access in the initial and in the last stages of the experiment. The test room was furnished with two desks for the participants, placed one in front of the other with a translucent white panel in between to block the view of the other participant, and located close to the north façade to avoid glare from the sun entering the room from the south opening. A neutral colour-scheme characterised the test room: grey floor (reflectance 27%), white walls, ceiling and door (reflectance 81–84%), grey furniture (reflectance 48–50%), and white blackout curtain (reflectance 60%). The test room was equipped with measurement devices for the indoor environment (i.e., temperature, light quantity, air velocity, relative humidity and CO_2_ content), continuously recording at 1-minute sampling rate. Figure 1 illustrates their location in the test room.

**Figure 2:**
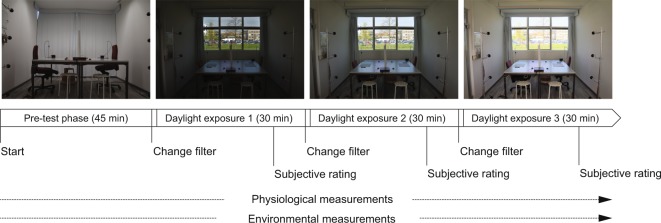
Experimental procedure. The order of daylight conditions presentation was counterbalanced across participants and temperature levels.

Figure 2 describes the experimental procedure. Two participants took part in each experimental session, which lasted approximately three hours. Participants were requested to comply to a pre-defined clothing ensemble, to ensure the same level of thermal insulation (0.7 clo)^43^. A pre-test phase of 45 minutes used for thermal and metabolic adaptation purposes, was followed by three identical phases in which participants were exposed for a maximum of 30 minutes to each illuminance level. The three illuminance levels were presented in a counterbalanced order across participants and temperature levels (i.e., the six possible combinations resulting from a different order of presentation of the three illuminance levels – 3!, were equally distributed in the three thermal conditions). In the pre-test phase, characterised by the use of electric light only (at a constant illuminance level of 400 lx at the desk level, correlated colour temperature of 3800 K), participants were informed about the experiment with an information sheet and a verbal explanation by the researcher, signed the consent form, wore the physiological measurement devices, filled in a personal information questionnaire and completed assigned paper-based office tasks. In the explanation of the experiment, a single blind procedure was followed to avoid to bias participants. They were told that the goal of the experiment was to understand the relationship between work-performance, physiological responses and different indoor conditions, without specifying if and in which way conditions would be changed. In each of the subsequent daylighting exposures, participants performed the same office tasks and were asked to reply to a questionnaire related to their perception of the indoor environment. During daylight exposures, participants were seated at the desks and were free to visually explore the room and the outside view, but their primary view direction was parallel to the north opening due to the configuration of the test room furniture and the written tasks they had to perform. Right after the pre-test and in between each daylight exposure, a 10-minute break allowed for the change of the filters on the glazing. During these breaks participants were blindfolded and were asked to sit still and relax by listening to music with headphones (both measures were taken to avoid that participants noticed the application of the filters on the glazing). The researcher was continuously present in the room during all experimental sessions to guide participants and to avoid changing the indoor environmental conditions by entering and exiting the room.

Experiments were performed during sunny days only (i.e., clear sky conditions) to guarantee a constant daylight level throughout each experimental session. Experimental sessions with unpredicted changing daylight conditions that resulted in variation of indoor illuminance levels during the experimental time (N = 19, equivalent to responses of 38 participants) were excluded from the analysis. The choice of the experimental sessions to include in the analysis was performed after the completion of each session with an analysis of the recorded horizontal illuminance levels to detect potential variations over the experimental time. For exploiting at best the daylighting hours, experiments were performed in both mornings (64%) and afternoons (30%), with experimental sessions starting from 8:30 to 9:00 and from 12:30 to 13:00, respectively. The study took place from October 2017 to December 2017 and from April 2018 to June 2018. Experiments were not conducted continuously due to weather conditions.

### Measurements

#### Subjective perception ratings

At the end of each daylight exposure, participants were asked to evaluate their perception of the indoor environment in terms of thermal perception and overall perception.

For thermal perception, four standardised thermal questions were used^48^, asking people to judge their personal thermal state (thermal sensation –asv, comfort and preference) and the thermal ambiance (thermal acceptability). The personal thermal state part was extended to three additional thermal sensation questions referring to specific body parts (hands, trunk and feet) and used seven-point and five-point categorical scales. The thermal acceptability question used a numerical visual analogue scale. As already anticipated, thermal sensation is seen differently from thermal comfort, preference and acceptability, the latter considered as thermal evaluations resulting from the thermal sensation^19^.

Two questions on overall comfort were asked at the beginning and at the end of each daylight exposure. First of all, similarly to the thermal comfort question, participants were asked to evaluate their overall comfort in terms of light, temperature, noise and air quality on a five-point categorical scale. Then, whenever they expressed dissatisfaction, they were asked to indicate the reason of their discomfort (adapted from Ackerly *et al*.^49^). All questions were in French and were asked in the same order, according to standards specifications^48^. Table 2 summarises the questions and the corresponding response scales.

**Table 2:** Thermal and overall perception questions and response scales.

	Question	Response scale
Thermal perception	Thermal sensation: How do you feel in this precise moment? I am…	Cold (−3), cool (−2), slightly cool (−1), neutral(0), slightly warm (+1), warm (+2), hot (+3)
	Thermal comfort: Do you find it:	Very comfortable (5), comfortable (4), slightly uncomfortable (3), uncomfortable (2), very uncomfortable (1)
	Thermal preference: In this moment, you would prefer to feel:	Much cooler (−3), cooler (−2), slightly cooler (1), no change (0), slightly warmer (+1), warmer (+2), much warmer (+3)
	Thermal sensation (body parts): How do you perceive the following parts of your body (hands, trunk and feet)	Cold (−3), cool (−2), slightly cool (−1), neutral (0), slightly warm (+1), warm (+2), hot (+3)
	Thermal acceptability: How do you judge this environment (local climate)?	Unacceptable (0) - Acceptable (100)
Overall perception	Overall comfort: How do you judge the global indoor environment (considering light, temperature, noise and air quality):	Very comfortable (5), comfortable (4), slightly uncomfortable (3), uncomfortable (2), very uncomfortable (1)
	Discomfort reasons: You have said that you are not comfortable with the global indoor environment. Which of the following has contributed to your discomfort?:	Visual conditions, thermal conditions, noise from the inside or outside the room, presence of other people in the room, poor air quality, lack of personal control, other

#### Physiological measurements

Together with subjective perception ratings, physiological responses to the thermal environment were recorded. Specifically, the skin temperature (SKT) of four body sites (i.e., neck, right scapula, right shin and left hand)^50^ was measured at 1-minute sampling rate with the wireless iButtons^®^ data loggers (DS1921H-F5, Maxim Integrated), sensors validated for measurements on the human skin^51,52^.

### Data processing and statistical analyses

SKT measurements were pre-processed prior to the statistical analysis. For each of the four measurements (SKT_*neck*_, SKT_*scapula*_, SKT_*shin*_ and SKT_*hand*_) an average value was calculated within the first five minutes and from fifteen to twenty minutes for each daylight exposure to observe the physiological responses during the adaptation time and right before the subjective perception questionnaire, respectively. A baseline value was calculated within the relaxation break prior to each daylight exposure for all participants to take into consideration individual differences. For each baseline value, the average was computed over a 5-minute period, after the first three minutes from the beginning of the relaxation time, to avoid including measurements possibly affected by the movement of participants.

Linear mixed models were used for the statistical analysis of subjective ratings and physiological responses to take into account the repeated measurements corresponding to each daylight exposure. Daylight and (design) temperature were modelled as fixed effects, together with their interaction. Participant ID codes were included in the model as random factors (random intercept model)^53^. Potential confounding factors were modelled as covariates of the model. These can be classified in three main groups. The first includes variables that have been identified to directly affect thermal perception or physiological responses, namely sex^54^ and body mass index – BMI^55^. The second consists of factors that changed during the experimental session or over days, namely the difference between the measured operative temperature and the design one – ∆T (the measured operative temperature itself was not included in the analysis as it would have resulted in a problem of collinearity, due to its close value to that of the design temperature, included as fixed effect), and the outdoor temperature calculated as running mean outdoor temperature according to the standard EN 15251^11^. Finally, the last group encompasses factors that were not balanced across participants (i.e., time of the day) or that were randomised across the experiments (i.e., order of daylight level presentation). In the analysis of the physiological measurements, baseline values were also considered as covariates. All subjective ratings were analysed separately after having been converted into numerical ordinal scales (whenever necessary). When the factor daylight was shown to have a significant main effect, post-hoc Tukey tests were conducted on all pairwise comparisons of the three daylight levels. The same analysis was conducted for temperature levels, when temperature resulted as a significant main factor. In the case of significant interaction between temperature and daylight, further analyses were conducted to consider only a few levels of each factor (e.g., only the low and the high illuminance levels) at each temperature level.

PMV index predicts the mean comfort response of a larger group of people according to physical and personal variables^5,43^. PMV values were calculated for each participant and in each daylight exposure according to environmental measurements (air temperature, mean radiant temperature –derived from the measured globe temperature, air velocity and relative humidity), clothing insulation (0.7 clo)^43^ and metabolic rate (1.2 met)^43^. PMV and asv were compared at each temperature level with the use of the non-parametric Wilcoxon signed-rank test (Matched-Pairs)^56^, following the Shapiro-Wilk normality test^57^. The two distributions were considered dependent as they report the evaluation (actual and predicted) of the same thermal environment. Cohen’s d was calculated to assess the magnitude of the difference between the two evaluations and should be interpreted using relevant thresholds (0.41 for small effect size, 1.15 for moderate effect size and 2.70 for strong effect size)^58^.

All statistical analyses were performed using R^59^ with the RStudio integrated development environment (RStudio Inc., Boston, MA, USA). Linear mixed models were performed with the *lmerTest* package^60^, while post-hoc comparisons were calculated using the *lsmeans* package applying the multiplicity adjustment Tukey’s HSD to control for false discovery rate^61^. PMV calculations were performed with the *comf* package^62^. All graphs were created with the *ggplot2* package^63^. The significance level for all analyses was set at 0.05.

## Results

First, the environmental data measured during the experiments are reported. Then, the results of the thermal and overall subjective perception ratings analysed with the linear mixed-models are described, followed by the results of the physiological responses. Finally, the comparison between PMV and asv is illustrated.

### Indoor environmental conditions

#### Thermal environment

Indoor temperature was measured at a distance of 50 cm from each participant and at four heights (0.1 m, 0.6 m, 1.1 m and 1.6 m), corresponding to ankle, body, head (sitting person) and head (standing person) levels according to the EN ISO 7726 standard^64^. Both air temperature and globe temperature were recorded, so as to also capture the temperature resulting from the radiation of the adjacent surfaces, air temperature and air velocity. Operative temperature values, calculated from the measured air and globe temperature^13,18^, were used in the analysis. The resulting values did in fact not differ too much from either globe or air temperature due to the nature of the radiant heating and cooling system and the very low air velocity: the mean absolute difference between globe and air temperature was 0.19 °C, while the root mean squared difference between globe and air temperature was 0.24 °C. Differences were on average larger at the lowest height given the proximity of the sensor to the radiant floor. For each participant, an average operative temperature was calculated over the four measurement heights.

**Figure 3:**
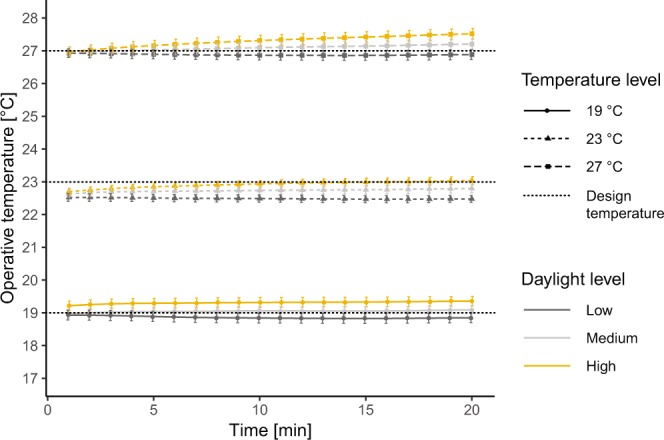
Average operative temperature in each temperature and daylight level combination.

Figure 3 illustrates the average operative temperature for the three thermal conditions investigated, in each daylight exposure. The design temperature is indicated with a black dotted line to observe the trends of the measured temperatures in each daylight conditions. In fact, the test room allowed the setting of a specific temperature, but the different filters applied on the glazing did slightly affect the indoor thermal environment due to changes in solar heat gains (i.e., the solar transmission was higher when the visible transmittance was higher). As a result, at the end of each daylight exposure the operative temperature was higher compared to the beginning, and a slightly higher operative temperature resulted under the high illuminance level, followed by the medium and the low ones. Table 3 reports the average operative temperature values for each design temperature level measured at 20 minutes after the beginning of each daylight exposure, when the differences in temperature values across daylight levels were larger. As anticipated, these temperature variations across daylight levels were considered in the statistical analysis with the inclusion, together with the design temperature levels (i.e., 19, 23 and 27 °C considered as nominal levels), of the covariate ∆T. The latter was calculated as the difference from the design temperature level (e.g., 23 °C) and the measured operative temperature (e.g., 22.8 °C) at 20 minutes after the beginning of each daylight exposure as this time corresponded to the start of the questionnaire.

**Table 3:** Average ± SD operative temperature in each temperature and daylight level combination (values in °C).

	Low	Medium	High
19 °C	18.85 ± 0.79	19.09 ± 0.71	19.35 ± 0.78
23 °C	22.48 ± 0.56	22.80 ± 0.71	23.04 ± 0.65
27 °C	26.88 ± 0.81	27.21 ± 0.86	27.52 ± 0.87

Other indoor parameters involved in the perception of the environment were constant across daylight and temperature levels (i.e., air velocity at 0.03 ± 0.005 m/s; CO_2_ content at 1115.2 ± 183 ppm) or changed only across temperature levels but within a range considered as comfortable (i.e., relative humidity: 56.8 ± 6.6% at 19 °C, 44.6 ± 8.9% at 23 °C and 42.3 ± 4.4% at 27 °C).

#### Visual environment

The visual environment was constant *within* each experimental session as only sunny days were retained for the analysis (i.e., the daylight illuminance did not change). This was possible as experimental sessions with unpredicted changes in sky conditions and in illuminance levels were not included in the analysis, as already anticipated. On the other hand, illuminance conditions were not exactly the same *across* experimental sessions (i.e., in different days). The recorded horizontal illuminance (calculated as the average of two values measured at the desk of each participant, on their right and left) at each exposure level can be summarised as follow:

- Low daylight illuminance: 136 ± 20 lx (min 90 lx, max 214 lx)
- Medium daylight illuminance: 608 ± 90 lx (min 432 lx, max 796 lx)
- High daylight illuminance: 1443 ± 183 lx (min 1049 lx, max 1929 lx)

The observed variations in daylight illuminance values, despite the inclusion of only sunny days in the analysis, are due to changes in the sun position across the year and the day, and the presence of haze and atmospheric turbidity^65^, conditions changing across experimental sessions (and not within them).

### Subjective perception ratings

**Figure 4:**
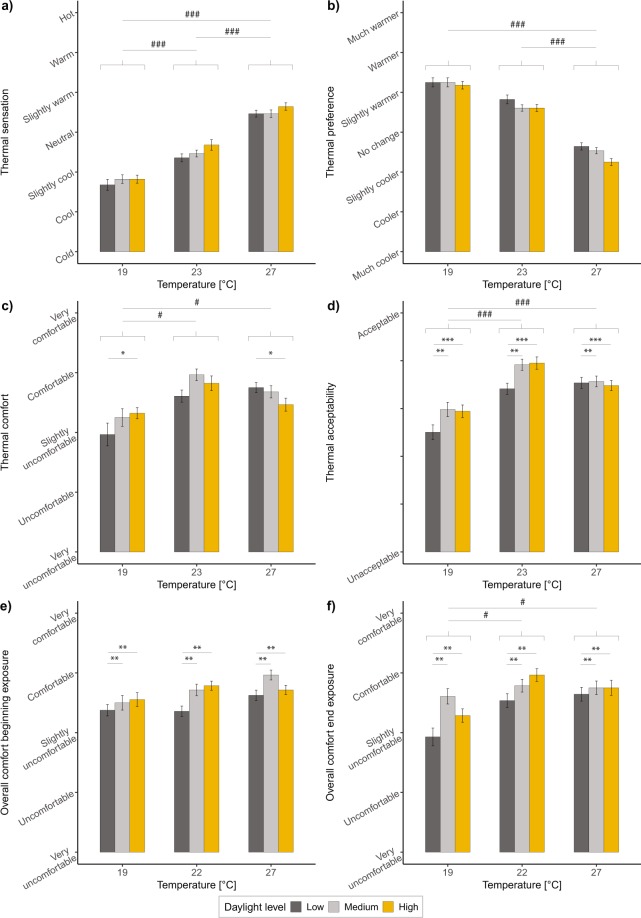
Subjective thermal and overall perception responses according to temperature and daylight illuminance levels. (**a**) Thermal sensation. (**b**) Thermal preference. (**c**) Thermal comfort. (**d**) Thermal acceptability. (**e**) Overall comfort (beginning of exposure). (**f**) Overall comfort (end of exposure). Significant effect of daylight levels with ^“*”^*p* < 0.05, ^“**”^*p* < 0.01, ^“***”^*p* < 0.001. The same effect is reported on all the bars for all the temperature levels whenever there is a main effect of daylight, otherwise separate effects of daylight at each temperature level are indicated (i.e., thermal comfort). Significant effect of temperature levels with ^“#”^*p* < 0.05, ^“##”^*p* < 0.01, ^“###”^*p* < 0.001.

Results related to thermal perception are discussed first, followed by those regarding overall perception. For the thermal perception analysis, each question is analysed individually and the main effect of daylight illuminance and its interaction with temperature are first reported. To check the validity of the experiment and the questionnaire used, the main effect of temperature (intended as the design factor with three experimental levels) is also described. For the overall perception analysis, main effects of both daylight illuminance and temperature are described, together with their interaction. Figure 4 reports the graphical outcomes of the two types of subjective ratings at each temperature level and for the three daylight illuminance levels.

#### Thermal perception

The analysis of thermal perception responses is divided according to the four questions investigated: thermal sensation (global and referring to the three body parts), thermal preference, thermal comfort and thermal acceptability.

Linear mixed model analyses showed that daylight illuminance did not influence thermal sensation, nor did interactions between temperature levels and daylight illuminance. The variations in thermal sensation votes across daylight illuminance levels that can be observed in Fig. 4a depended on the temperature differences across the illuminance levels, illustrated in Fig. 3. In the model, this difference is considered with the inclusion of ∆T, a factor that was shown as being significant for thermal sensation based on the statistical analysis (*F*(1,245) = 11.09, *p* = 0.001), with larger ∆T associated to higher or lower thermal sensation votes compared to those associated to smaller ∆T (i.e., the higher the measured temperature, the higher the thermal sensation vote; the lower the measured temperature, the lower thermal sensation vote). Nevertheless, it must be remarked that, although the average temperature differences between daylight illuminance levels were similar (see Fig. 3 and Table 3), the average differences in thermal sensation votes were not as constant, especially at 19 °C and at 27 °C. At 19 °C, the average thermal sensation votes were comparable under the medium and the high illuminance levels, but they were lower under the low illuminance one. At 27 °C, the average thermal sensation votes were comparable under low and medium illuminance levels, but they were higher under the high level. Although not significant, these results indicate a tendency of participants to feel cooler at low temperatures under low illuminance level compared to medium and high illuminances, and to feel warmer at high temperatures under high illuminance level compared to low and medium. As expected, temperature was also a significant factor for the determination of the thermal sensation (*F*(2,245) = 74.25, *p* < 0.001), with participants expressing to be between cool and slightly cool at 19 °C (*M* = −1.3, *s*.*e*.*m*. = 0.84), between slightly cool and neutral at 23 °C (*M* = −0.5, *s*.*e*.*m*. = 0.84) and between neutral and slightly warm at 27 °C (*M* = 0.5, *s*.*e*.*m*. = 0.75). These differences were shown to be significant following post-hoc pairwise comparisons of all possible combinations. The analysis performed for the thermal sensation of hand, feet and trunk reported the same results as for the global thermal sensation, confirming the results previously described.

Similarly to thermal sensation, thermal preference results (Fig. 4b) were not affected by daylight illuminance either, nor by its interaction with temperature. Only ∆T and temperature levels were shown to have significant effects (*p* = 0.001 and *p* < 0.001, respectively). Participants expressed a preference for a slightly warmer-warmer environment at 19 °C, no change-slightly warmer at 23 °C and no change-slightly cooler at 27 °C.

Results are different for both thermal comfort and thermal acceptability, with daylight illuminance significantly affecting such thermal evaluations. First of all, the interaction between daylight and temperature was a significant factor for thermal comfort, in particular when only the low and the high daylight illuminance conditions were investigated at 19 °C and 27 °C (*F*(1,107) = 6.73, *p* = 0.012), as well as at 23 °C and 27 °C (*F*(1,102) = 6.14, *p* = 0.016). As can be seen in Fig. 4c, and following analyses at each temperature level with only the low and high daylight illuminance levels, the thermal environment was less comfortable under the low illuminance compared to the high one at 19 °C (estimated difference of 0.48, *p* = 0.03 after post-hoc test), whereas it was less comfortable under the high illuminance level compared to the low one at 27 °C (estimated difference of 0.28, *p* = 0.03 after post-hoc test). Differences between low and high daylight illuminance levels were not significant at 23 °C, thermal condition in which the medium illuminance appears as the most comfortable one (Fig. 4c). Temperature resulted as a significant main factor for thermal comfort, with post-hoc analyses showing significant differences between 19 °C and 23 °C (*p* = 0.01), and between 19 °C and 27 °C (*p* = 0.01), with people being always less thermally comfortable under 19 °C.

The interaction term was not significant for thermal acceptability responses. On the other hand, daylight illuminance was a significant factor (*F*(2,247) = 6.4, *p* = 0.001), with a less acceptable thermal environment under the low illuminance level compared to both the medium and the high illuminances (*p* = 0.004 and *p* = 0.009, respectively). Despite the lack of interaction with temperature, it was possible to see that this result specifically occurred at 19 °C and 23 °C, temperature levels considered as cool-slightly cool and slightly cool-neutral, respectively (Fig. 4d). At 27 °C, thermal acceptability responses were equal under all the light levels. As for thermal comfort, thermal acceptability votes were affected by temperature (*F*(2,247) = 8.74, *p* < 0.001), with participants less accepting of the thermal environment under 19 °C compared to 23 °C (estimated difference of 19.01, *p* < 0.001 after post-hoc analyses) and under 19 °C compared to 27 °C (estimated difference of 14.37, *p* = 0.009 after post-hoc analyses).

∆T, calculated as the difference from the measured indoor temperatures and the three temperature levels (i.e., 19, 23 and 27 °C), had a considerable effect on all the thermal perception responses, except for thermal acceptability. As explained in more detail for the thermal sensation responses, the inclusion of the factor ∆T increased the accuracy of the evaluation as it followed the direction of the model (e.g., higher measured temperatures resulted in “more extreme” thermal sensations).

Sex and order of daylight illuminance levels were significant factors for thermal sensation and preference. The running mean outdoor temperature substantially affected thermal sensation and comfort. Thermal comfort was also influenced by participants’ BMI and morning and afternoon exposures.

#### Overall perception

**Figure 5:**
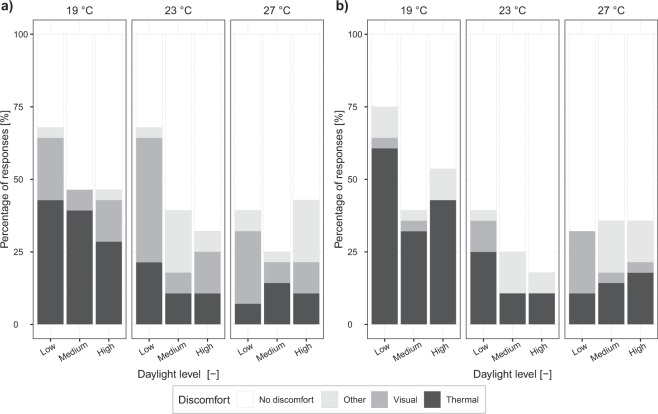
Reasons of overall discomfort at the beginning and at the end of each daylight exposure.

The overall comfort perception was evaluated at the beginning (Fig. 4e) and at the end (Fig. 4f) of each daylight illuminance exposure. At the beginning of the exposure, overall comfort perception was significantly affected by daylight illuminance levels (*F*(2,247) = 7.40, *p* < 0.001) (Fig. 4e), with the low illuminance level resulting in the lowest overall comfort compared to the medium level (estimated difference of 0.27, *p* = 0.001 after post-hoc analyses) and to the high level (estimated difference of 0.23, *p* = 0.008 after post-hoc analyses). The main effect of temperature was only marginal at the beginning of the exposure (*F*(2,247) = 2.87, *p* = 0.06). On the other hand, at the end of the exposure (Fig. 4f), both daylight and temperature had a significant main effect (*F*(2,246) = 7.25, *p* < 0.001 and *F*(2,246) = 5.27, *p* = 0.006, respectively). The increasing effect of temperature on overall comfort at the end of the exposure can be seen especially for responses at 19 °C, which were significantly lower in comparison to those under the neutral and the high thermal conditions (estimated difference of 0.45, *p* = 0.001 and of 0.42, *p* = 0.002, respectively, after post-hoc analyses). Figure 5 reports the reasons of overall discomfort at the two exposure times. At the beginning (Fig. 5a), when the indoor temperature was still not affected by the incoming sun from the window (hence, no big real temperature differences were present across daylight levels), more complaints about the thermal environment were reported under the low illuminance level, followed by the medium and high levels, especially at the temperature levels considered as slightly cool (i.e., 19 °C and 23 °C). Complaints about the visual environment decreased at the end of the exposure compared to the beginning, as participants indicated the thermal environment as the reason of overall discomfort more often. This is an interesting fact because, even though overall comfort responses at the end of the exposure depended on both daylight and temperature levels as previously reported (Fig. 4f), participants mainly indicated “thermal” as the reason of their dissatisfaction, rather than both “thermal” and “visual”.

### Physiological responses: skin temperature

Daylight was never a significant factor for the skin temperature measurements in any of the four body locations, considering the results within both the first five minutes and between fifteen and twenty minutes (the time right before the subjective perception questionnaire). As expected, measurements were mainly affected by temperature and by the baseline values (*p* < 0.001 in both cases).

### PMV and thermal sensation vote comparison

**Figure 6:**
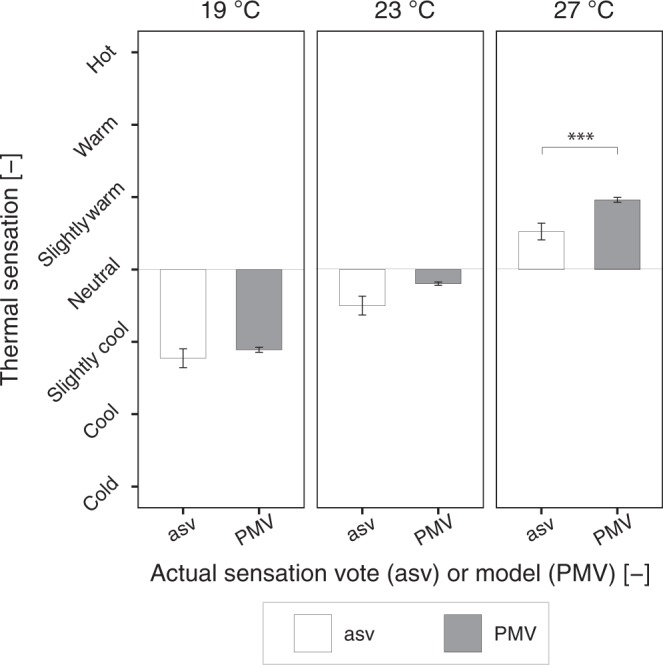
Comparison between actual thermal sensation vote and PMV at the three temperature levels (mean ± s.e.m.).

**Figure 7:**
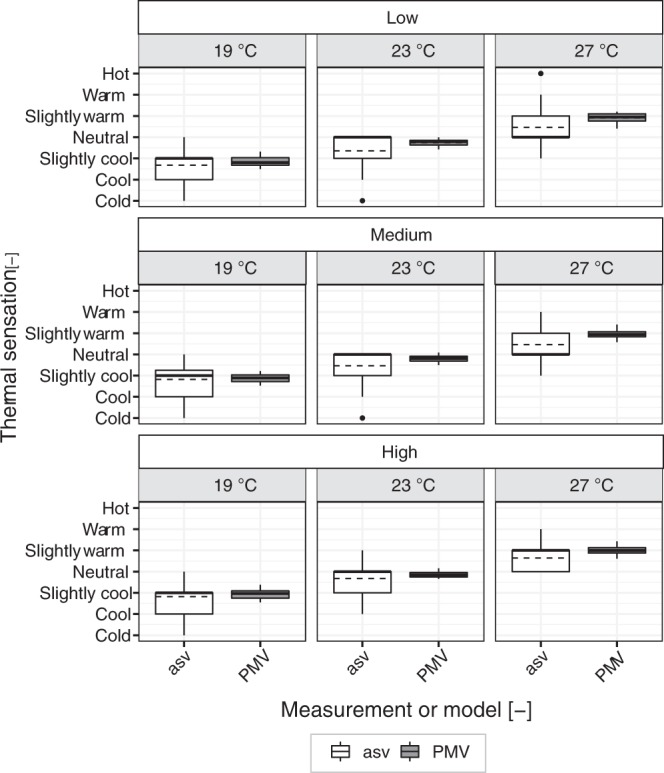
Boxplot comparison between actual thermal sensation vote and PMV at the three temperature levels and three daylight conditions (the dark line indicates the median and the dashed line illustrates the mean of the distribution of votes).

Thermal sensation vote (asv = actual sensation vote) of participants were considered binned in each temperature level independently of the daylight illuminance level and were compared with the PMV values, calculated from the measured physical conditions (Fig. 6). Results show that the average values of asv differed significantly from the PMV ones, but only at the warmer thermal condition (27 °C) according to the Wilcoxon signed rank test (*p* < 0.001 and *d* = 0.61). At this temperature level, the asv vote was substantially lower (*M* = 0.52, *SD* = 0.75) compared to the PMV (*M* = 0.96, *SD* = 0.21). At 23 °C the difference resulted marginally significant (*p* = 0.053 and *d* = 0.37), whereas at 19 °C no significant difference was reported by the test (*p* = 0.42). The larger standard deviation of the reported asv votes compared to the calculated PMV values that can be observed in Fig. 6, was due to the inclusion of results in different daylight illuminance levels that, as reported before, led to changes in thermal perception ratings. A comparison of asv and PMV votes in each illuminance-temperature combination is illustrated in Fig. 7. In Fig. 7, it is possible to see that the overestimation of the PMV in comparison to asv at 27 °C is particularly visible under the low and the medium daylight illuminance levels. Under the high illuminance level at 27 °C, the difference between asv and PMV is smaller. This result further corroborates the effect of daylight illuminance levels on subjective thermal perceptions of people.

## Discussion

This study evaluated the effect of light quantity (i.e., illuminance levels) on thermal responses (i.e., subjective thermal perception and skin temperature) with the use of daylight as the only source of light and in a laboratory setting, investigating such effect in various thermal conditions to be able to capture interaction effects. With this type of experimental design, it was also possible to evaluate the combined effect of daylight illuminance and temperature levels on overall comfort perception. Due to the use of daylight, it is difficult to compare results with those of past studies, as these latter were conducted with electric light only. Moreover, as discussed in the introduction section, results of past investigations were contradicting for both skin temperature measurements and subjective thermal perception ratings. Different results have been explained in terms of experimental design features such as the timing and duration of the light exposure, as well as in terms of light intensity used. We also suggested that two additional factors should be considered in the analysis, namely whether the time of the light exposure corresponds to that of the thermal measurements, and the type of thermal environment to which people are exposed (i.e, static vs. dynamic). Considering these factors, the present study investigated the cross-modal effects of three levels of daylight illuminance (low, medium and high) on thermal responses, in three static thermal conditions, as well as the combined effect of daylight and temperature levels on overall perception, with experiments performed during daytime and for a short exposure time (i.e., less than 30 minutes). Responses of people were collected while they were exposed to both visual and thermal stimuli.

Results indicate that daylight affected the thermal perception of people but not their physiological responses in terms of skin temperature. The effect of daylight on thermal perception differed according to the thermal condition, and the type of thermal perception investigated. With reference to this latter factor, it was possible to see that daylight did not affect the thermal sensation (global and of the body parts) and thermal preference responses, while it substantially influenced thermal comfort and thermal acceptability responses. The different outcomes of the thermal perception evaluations support the notion that sensation, preference, comfort and acceptability are different constructs that measure various aspects of the subjective thermal perception^19,20,48^. Based on the results of this experiment, it is plausible that daylight lies amongst the factors affecting the thermal *evaluation* of the indoor environment. The fact that daylight levels did not affect physiological responses, corroborate the hypothesis that, after a short exposure time, daylight could influence thermal perception only on a psychological level. Moreover, it strengthens the relationship between physiological responses and thermal sensation, as neither of them was substantially affected by daylight. The influence of daylight on thermal evaluation is of interest in both research and practice. First of all, daylight should be considered as an influencing factor in thermal comfort research (hence it should be controlled or at least measured and reported). In practice, a building’s operation in terms of temperature setting could be modulated by the visual conditions (in existing buildings) or building characteristics could be designed according to the thermal environment, to guarantee occupants’ comfort.

Results about the overall perception showed that both temperature and daylight were important factors for the evaluation of the indoor environment. In particular, the thermal environment became more important at the end of the exposure, while the visual environment lost part of its effect, indicating a probable adaptation to light levels within each daylight illuminance exposure. At the end of each daylight exposure, the majority of people indicated thermal factors as the principal source of overall discomfort despite being substantially affected by both temperature and daylight levels. This result further corroborates the psychological effect of daylight on thermal perception and the fact that daylight illuminance levels might affect the thermal perception of people in an indirect way.

Confirming the hypothesis of Veitch *et al*.^42^, according to which the thermal environment in a naturally-lit space could be more tolerated than what is predicted by the PMV, the thermal sensation of participants (asv) was lower compared to the calculated PMV based on the measured indoor conditions. Results, however, differed according to the thermal environment considered: the average difference between the asv and the PMV votes was marginal at 23 °C and substantial only at the highest temperature level investigated (27 °C), resulting in less warm asv compared to the PMV calculated with the heat balance model. This result indicates an overestimation of PMV in warm thermal conditions in comparison with the thermal sensation votes expressed by participants. Several other studies reported the overestimation of the PMV^6,7,17,66,67^, especially at higher thermal conditions (e.g., higher than 28 °C)^17^. Results of these studies (except those of Yang *et al*.)^67^ were derived from field studies and the difference between the actual and the calculated thermal sensation votes was mainly explained in terms of *behavioural thermal adaptation* (e.g., participants could adapt to the thermal environment by opening and closing doors and windows or changing their clothing, in contrast to the lack of control and adaptation in controlled experiments). In the study performed in the controlled environment^67^, the authors also explain results in terms of thermal adaptation, but this time with reference to the *acclimatization* of their participants to the hot-humid climate in which they were living (Chongqing, China), explaining that habituations neutralised their thermal sensation due to moderated thermal sensibility of the skin. Considering that the current study was conducted in a controlled experimental setting in which participants could not adapt in any way to the thermal environment and that the majority of participants were not from a hot-humid climate (86% were from Europe), it is plausible that the lower thermal sensation reported by people was determined solely by the *presence of daylight in the room*, considering that this might be the predominant difference between the current experiment and the controlled one performed for the calculation of the PMV^5^. An adaptive process in terms of *expectation* could have occurred. In other words, in a warm environment, people could be more forgiving of the thermal conditions because they would expect a warm environment due to the presence of daylight (and hence of solar heat gains). The heat balance model, derived from experimental investigations in a controlled environment with the use of electric light, could not capture the psychological effect of daylight, resulting in the overestimated PMV values at higher temperature levels. This result is of more than scientific interest as it could have practical consequences given the extended presence of daylight in buildings. Its implementation could lead to the reduction of unnecessary cooling in naturally-lit warm environments, resulting in substantial reduction in energy consumption. Moreover, considering that the largest differences between asv and PMV values were observed for the low and the medium illuminance levels (conditions that could be achieved limiting the sun penetration in indoor spaces), the downside of additional solar heat gains due to the presence of daylight could be avoided. To give a general idea of the magnitude of the possible energy savings, we calculated the change in operative temperature resulting from the change in mean thermal sensation between the asv and PMV in the warm thermal condition (Fig. 6). Calculations, performed with the Berkeley CBE thermal comfort tool^68^ using the actual conditions in the room (metabolic rate typical for office work – 1.2 met, clo level provided to participants – 0.7 clo, measured air velocity – 0.01 m/s, and measured relative humidity – 42.3%), indicated that participants in the warm condition expressed a thermal sensation vote as if they were exposed to 25.5 °C, 1.7 °C less than the average measured operative temperature in the warm condition, equal to 27.2 °C. Considering that the experiment was not designed for this specific investigation (i.e., comparison of the thermal perception of people exposed to electric lighting or to daylight) and that differences were observed solely at one temperature level experienced only by few participants (i.e., 28 people), it is necessary to perform additional investigations with experimental studies designed for this purpose to be able to validate the hypothesis that only the presence of daylight could decrease the cooling needs in warm environments. Collecting information about the visual environment besides the standard thermal comfort measurements in thermal comfort studies^69^, could further help the understanding of the cross-modal effect of daylight on thermal responses and could validate the proposed hypothesis.

As a general remark, it must be noted that the results obtained in this study refer to the specific conditions investigated (i.e., controlled experimental conditions, to which participants were exposed for a short exposure time). Considering the psychological nature of the reported findings, results might differ for longer exposure time and could be masked (or enhanced) by other factors that could be present in real buildings. Moreover, cultural aspects might also affect the results, as well as climatic and seasonal differences. Further investigations are suggested to expand our understanding on the effect of daylight illuminance levels on thermal responses.

## Conclusions

This paper describes a controlled experiment investigating the impact of three daylight illuminance levels (low, medium and high) on thermal responses in three different thermal conditions (19, 23 and 27 °C), as well as that of the combined presence of daylight and temperature levels on overall comfort perception. Results indicate that a short exposure time (i.e., less than 30 minutes) to daylight during daytime influenced the subjective thermal perception of people while they were exposed to static thermal conditions. Their skin temperatures measured in four body locations were however not affected by daylight but just by the temperature levels. These results indicate that cross-modal effects of daylight on thermal responses occurred, but only at a psychological level rather than at a physiological one.

This conclusion is corroborated by results in terms of subjective thermal perception, as daylight affected only thermal evaluations (thermal comfort and acceptability) but not thermal sensation, a subjective perception usually attributed to physiological changes. In particular, participants reported being more thermally comfortable in a high illuminance level compared to a low one in a cold condition (19 °C), whereas in a slightly warm environment (27 °C), participants indicated being more thermally comfortable under the low illuminance level compared to the high one. Moreover, in terms of thermal acceptability responses, the thermal environment was less acceptable under low illuminance condition compared to both the medium and the high levels. These cross-modal effects of daylight on subjective thermal perception were also confirmed by responses about overall perception.

Finally, only in the warm thermal condition, the thermal sensation reported by participants was lower than that predicted by the heat balance model according to the indoor parameters measured during the experiment (PMV values), especially under low and medium illuminance levels. In the warm thermal condition, it was calculated that the reported average thermal sensation corresponded to a perceived operative temperature of 1.7 °C lower than the average measured temperature. It was hypothesised that a thermal adaptation in terms of expectation might have occurred, explaining such result: people could be more forgiving of a warm environment in a naturally-lit space compared to the same thermal condition in the presence of electric light only. This result highlights the psychological nature of the cross-modal effect of daylight on thermal perception and confirms the importance of considering visual conditions in thermal comfort evaluations.

The findings presented here provide the first evidence that daylight can affect human subjective thermal perception from a psychological point of view and that results depend on the thermal environment to which people are exposed to. Specifically, it has been shown that, despite the fact that more daylight is associated to more sunrays and subsequent heat gains (a physical phenomenon impossible to uncouple), interactions between daylight and the thermal environment can occur on a perceptual level. Findings open new prospects in human thermal comfort research, helping shed more light on the factors affecting adaptive processes in indoor environments. Moreover, these findings highlight the fact that temperature and daylight illuminance conditions should be tuned and controlled through building design and operation to guarantee occupants’ thermal comfort. In conclusion, daylight should be considered as a factor affecting thermal perception in both practice and research, and visual characteristics of the indoor environment should be recorded and considered together with the thermal factors in future research investigations and design projects.

## Supplementary information

LaTeX Supplementary File

LaTeX Supplementary File

LaTeX Supplementary File

LaTeX Supplementary File

## Data Availability

The datasets generated during and/or analysed during the current study are available from the corresponding author on reasonable request.
